# Will it blend? Exploring the viscoelastic characteristics of P3HT-polyborosiloxane blends towards flexible electronic materials†

**DOI:** 10.1039/d4lp00163j

**Published:** 2024-10-10

**Authors:** Peter A. Gilhooly-Finn, Megan M. Westwood, Bob C. Schroeder

**Affiliations:** a Department of Chemistry, University College London 20 Gordon Street London WC1H 0AJ UK of Great Britain and Northern Ireland p.finn@ucl.ac.uk b.c.schroeder@ucl.ac.uk; b Department of Chemistry and Chemical Engineering, Chalmers University of Technology 41296 Göteborg Sweden

## Abstract

Blending organic semiconducting polymers with elastomeric materials has been shown to be a successful method for improving the flexibility of wearable electronics. One such elastomer that has not been readily explored in combination with an organic semiconducting polymer is polyborosiloxane (PBS). PBS shows remarkable viscoelastomeric properties, due to the borate ester groups that crosslink the siloxane backbones, demonstrating a dynamic covalent crosslinking mechanism. The detailed study presented here showcases the properties of two different PBS elastomers and the effect of blending a well-known organic semiconducting polymer, poly(3-hexylthiophene) (P3HT). Compatibility studies showed that one elastomer blends more favourably than the other due to differences in the crosslinking density leading to the formation of P3HT crystallites within the blend. The viscoelastic properties of the PBS : P3HT blends are studied through detailed rheological experiments and the relaxation processes are discussed.

## Abstract 2

This study examines how blending P3HT with polyborosiloxane affects its mechanical and thermal properties, focusing on the crosslinking density and rheological behaviour.

## Graphical abstract

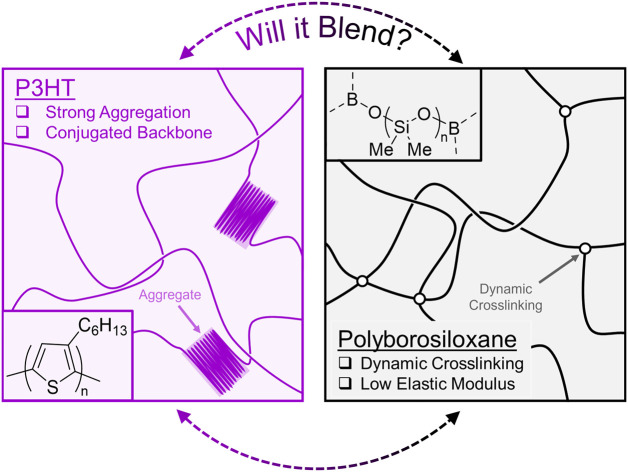

## Introduction

Flexible electronics are emerging as a research priority as electronic materials are increasingly integrated into wearable devices for bioelectronic monitoring and sensing applications.^1^ Semiconducting polymers have recently shown great potential in wearable applications compared to their inorganic counterparts, due to their chemical tuneability, inherent flexibility and light weight nature.^2,3^ Semiconducting polymers utilise π-conjugation along the polymer backbone to allow delocalised charge carriers to travel between electrodes in a device; however, they still exhibit a high elastic modulus when compared to the most flexible parts of skin (MPa *vs.* kPa).^4,5^ Many approaches have been successfully carried out to circumvent this issue,^6^ and one proven strategy is blending the semiconducting polymer with an elastomeric polymer that exhibits a lower elastic modulus.^7^ Notable examples are blends with polymer systems such as cross-linked poly(dimethylsiloxane) (PDMS),^8,9^ paraffin,^10^ poly(methyl methacrylate) PMMA,^11^ poly(styrene-ethylene-butylene-styrene) (SEBS),^12,13^ and butyl rubber.^14^ The best performing blends that exhibit high charge carrier mobility when subjected to high strain show large phase separation due to incompatibility between the two polymer systems. In fact, the phase separation induces the formation of a nanoconfined semiconducting polymer that has been shown to improve charge transport and device stability.^15^ Vertical and lateral phase separation between an organic semiconductor and insulator polymer is also a method researchers use to improve conduction pathways between electrodes in field effect transistors and sensing applications.^16^ Furthermore, supramolecular chemistry has been exploited when blending semiconducting polymers and elastomers as these dynamic interactions can assist in strain energy dissipation.^17^ Yet, the miscibility between semiconducting polymers and the elastomers that exhibit supramolecular chemistry has not been extensively studied.

**Fig. 1:**
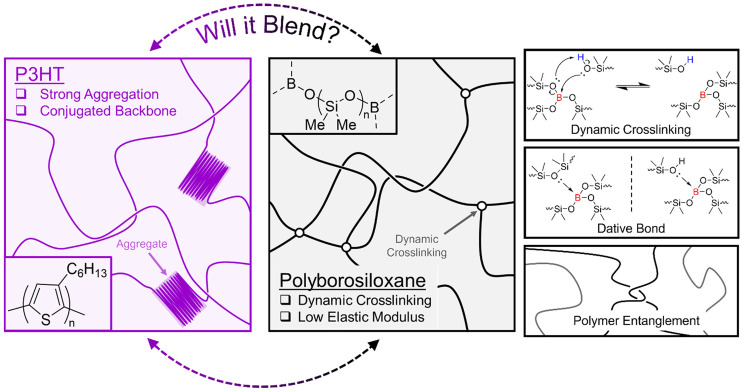
Diagram depicting the structure of P3HT (pink) and polyborosiloxane (black) and the different supramolecular interactions associated with polyborosiloxane.

One polymer of interest that exhibits supramolecular chemistry is the viscoelastomer polyborosilioxane (PBS) (Fig. 1). PBS contains a siloxane backbone crosslinked *via* boron ester groups and its ability to exhibit solid–liquid like properties arises from the formation of dynamic boron crosslinking sites. Currently, the dynamic bonding mechanism is widely accepted to arise from dative bonds between the empty p-orbital on the B atom and lone pair of electrons on any O atom; however, reports of a dynamic covalent bonding system have recently been published.^18,19^ As discussed in detail in the review by Drozdov *et al.*, the dynamic bonding interactions of PBS have enabled its use in applications such as shock protection and rheological fluids.^20^ Additionally, recent studies of blending PBS with electronic materials have enabled its use in sensors and conductors.^21–25^ Therefore, this study aims to widen the breadth of supramolecular polymer blends by showcasing the effects of blending an organic semiconducting polymer with PBS.

We chose the widely studied poly(3-hexylthiophene) (P3HT) as the organic semiconducting polymer. Not only does P3HT allow charge transport through its conjugated polythiophene backbone, but the addition of hexyl side chains confers it with excellent solubility in organic solvents such as chloroform and chlorobenzene (Fig. 1). P3HT is a well-studied semiconducting polymer and as such perfectly suited for a fundamental blending study with PBS. To gain deeper insights into the effects of supramolecular entanglements and crosslinking density within PBS, two different PBS polymers were synthesised. By performing a condensation reaction between a low and high molar mass hydroxy terminated PDMS (PDMS-OH) and boric acid, the corresponding low (LPBS) and high molar mass (HPBS) polyborosiloxanes were obtained. The molar masses of the two different PDMS-OH polymers were chosen with respect to the critical molar mass (*M*_c_) of linear PDMS (24.5 kg mol^−1^).^26^ LPBS was synthesised from a hydroxy-terminated PDMS with a molar mass lower than the *M*_c_ value, whereas HPBS was synthesised from PDMS-OH with a molar mass exceeding *M*_c_. The effect of this led to two different elastomeric networks which were studied using FTIR, differential scanning calorimetry (DSC), thermogravimetric analysis (TGA) and rheology. By obtaining the Hansen solubility parameters of P3HT and the PBS elastomers, the compatibility was theoretically deemed to be sufficient to produce a homogeneous blend. Using a combination of optical, thermal, and mechanical characterisation tools, the effects of blending P3HT into the two different PBS systems at concentrations between 0.1 and 10 wt% are discussed.

## Experimental

### Materials

Poly(3-hexylthiophene) was synthesised according to previous literature.^27^ The regioregularity was estimated to be 95% *via*^1^H NMR and the number-average molar mass (*M*_n_) and dispersity (*Đ*), determined *via* SEC (Size exclusion chromatography) in chlorobenzene at 80 °C, are 58 kg mol^−1^ and 2.0, respectively. The low and high molar mass hydroxy-terminated polydimethylsiloxanes (LPDMS-OH and HPDMS-OH) were purchased from Alfa Aesar and Sigma-Aldrich, respectively. Boric acid (B(OH)_3_) was purchased from Sigma-Aldrich and dried at 120 °C under vacuum before use. All solvents were purchased from Honeywell or Fisher. All chemicals were used as received unless otherwise stated.

### General experimental details

^1^H and ^11^B Nuclear Magnetic Resonance (NMR) spectroscopy methods were carried out on either a Bruker Avance III 400 and Bruker Avance Neo 700, respectively. Size exclusion chromatography (SEC) was performed on a Shimadzu LC-2030c system, run at 1 mL min^−1^ with chlorobenzene (80 °C) as the mobile phase and a mixed bead polystyrene gel (PLgel MIXED-B, supplied by Agilent) as the stationary phase. The sample was detected using an RID-40A detector and the molar masses were calibrated to known polystyrene standards (Agilent EASICAL PS-1). Fourier Transform Infra-Red (FTIR) spectroscopy was carried out on a Bruker Platinum ATR. Thermogravimetric analysis (TGA) was carried out on a TA Instruments TGA 5500 under a N_2_ atmosphere and a heating rate of 10 °C min^−1^. DSC was carried out on a TA Instruments DSC2500 with a TA LN2P Pump attached. DSC measurements were carried out at a heating rate of 10 °C min^−1^ under a He atmosphere. Rheology analysis was carried out at 25 °C using a Peltier heater on a Bholin Gemini rheometer using a 20 mm parallel plate with a gap size between 100 and 400 μm. Solution UV-vis absorbance spectroscopy was carried out on a Shimadzu UV-3600i Plus in chlorobenzene with increasing concentrations of polyborosiloxane (0–99.9 mg mL^−1^) and a constant concentration of P3HT (0.1 mg mL^−1^). A quartz cuvette of path length 1 mm was used.

### Synthesis of PBS

Two different polyborosiloxanes were synthesised, LPBS using a low molar mass PDMS-OH (*M*_n_ = 4.6 kg mol^−1^; *Đ* = 2.1) and HPBS employing a higher molar mass PDMS-OH (*M*_n_ = 40.0 kg mol^−1^; *Đ* = 2.1). Apart from the different molar mass PDMS-OH precursors, the general synthesis of the two polyborosiloxanes was identical and adapted from the literature.^28^ PDMS-OH (20.0 g, 270.3 mmol) was dissolved in toluene (250 mL) in a two-necked round bottom flask attached with Dean–Stark apparatus, a condenser and an internal thermometer. Boric acid was added (2.0 g, 32.3 mmol) and the suspension was stirred vigorously for 10 minutes at room temperature to ensure that boric acid was fully dispersed. Then the reaction mixture was heated to reflux, and the temperature was monitored and kept between 110 and 115 °C for 3 days. After cooling to room temperature, the toluene was removed under reduced pressure and the resulting viscous liquid further dried in a vacuum oven at 40 °C overnight to give the crude PBS polymer as a cloudy viscous oil. The crude PBS was redissolved in hexane (∼350 mL) using a combination of stirring, heating, and ultrasound sonication. The resulting solution was filtered through a 0.45 μm PTFE syringe filter to remove any excess and unreacted boric acid. The hexane was removed under reduced pressure and the purified polymer dried in the vacuum oven at 120 °C overnight to give PBS as a clear solid. The PBS was stored under vacuum between measurements and then heated to 120 °C under vacuum prior to any measurements to remove any residual moisture.

### Blending method

PBS (250 mg mL^−1^) and P3HT (25 mg mL^−1^) were dissolved separately in chlorobenzene at 80 °C under stirring for 3 h, before being combined in ratios of PBS : P3HT 900 : 100, 950 : 50, 990 : 10, 995 : 5 and 999 : 1 (w/w). The solutions were stirred overnight at 80 °C to ensure full mixing. After the magnetic stirrer bars were removed, they were placed in a vacuum oven at room temperature for 4 h, then at 70 °C for 2 h, and then at 120 °C overnight to dry to give a dark brown solid. The resulting polyborosiloxane : P3HT blends were stored under vacuum between measurements and then heated to 120 °C under vacuum prior to any measurements to remove any residual moisture.

## Results and discussion

**Fig. 2:**
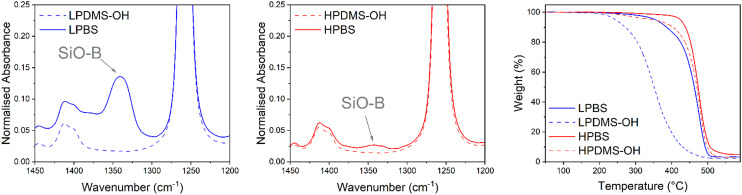
(Left & middle) Region of the FTIR spectrum showing the SiO–B stretch for LPBS (blue) and HPBS (red) compared to LPDMS-OH (dashed blue) and HPDMS-OH (dashed red). Each spectrum is normalised to the Si-CH_3_ stretch at 1258 cm^−1^. (Right) TGA thermogram of LPBS (blue), LPDMS-OH (dashed blue), HPBS (red) and HPDMS-OH (dashed red) recorded at 10 °C min^−1^ under a nitrogen atmosphere.

The reaction to produce the PBS elastomers used in this study from the precursor PDMS-OH polymers proceeds *via* a condensation reaction with boric acid, forming new B–O–Si bonds (Scheme S1†). To remove the water formed and drive the reaction to completion, Dean–Stark apparatus was used in conjunction with toluene–water azeotropic chemistry. An excess of boric acid was used to terminate all hydroxy groups on linear PDMS-OH. Unreacted boric acid was removed by redissolving the crude PBS elastomer in hexanes and filtering through a 0.45 μm PTFE syringe filter. Liu *et al.* studied in detail the effects of reaction temperature on PBS formation, and demonstrated significant chain scission at 200 °C, leading to a dramatic molar mass decrease after 1 hour of reaction time and unpredictable crosslinked structures.^29^ Using a lower reaction temperature (110 °C) should lead to a more defined elastomer with predictable mechanical properties.^28^ Two different PBS batches were synthesised from a low and high molar mass linear PDMS-OH (named LPDMS-OH and HPDMS-OH, respectively) precursor. The differences in number average molar mass (*M*_n_) were confirmed by size exclusion chromatography (SEC) in chlorobenzene and found to be 4.6 and 40.0 kg mol^−1^, respectively (Fig. S4 and Table S1†). According to Fetters *et al.*, the HPDMS-OH molar mass is above the *M*_c_ value of linear PDMS (24.5 kg mol^−1^), where polymer entanglements will largely contribute to a higher viscosity.^26^ The resulting polyborosiloxanes synthesised from LPDMS-OH and HPDMS-OH are named LPBS and HPBS, respectively. To assess whether the condensation reaction between the linear PDMS-OH and boric acid was successful, FTIR spectroscopy was carried out on the resulting PBS samples after being dried at 120 °C under vacuum overnight (Fig. 2). Compared to PDMS-OH, both the LPBS and HPBS samples showed a new peak at 1340 cm^−1^ assigned to the SiO–B stretch observed in the literature and according to Kurkin *et al.* is due to a trigonal planar B(OR)_3_.^19,30^

When the spectra are normalised to the peak at 1258 cm^−1^, which relates to the Si-CH_3_ stretch on the PDMS backbone, the relative intensity of the peak for LPBS compared to HPBS is an order of magnitude higher (0.14 *vs.* 0.03) indicating a larger number of end group B(OSi)_3_ crosslinking sites. Alongside the more intense SiB–O stretch, a small broad peak at 3210 cm^−1^ was also observed for LPBS which was assigned to O–H stretches, suggesting that even drying at 120 °C under vacuum does not lead to complete condensation or the presence of mono and di-borate esters (Fig. S5†). However, the intensity of this stretch is very low compared to the intensity of the SiO–B stretch, suggesting only a small number of terminal O–H bonds. To further estimate the boron content in the different PBS samples, ^11^B NMR was carried out by dissolving approximately 30 mg of each PBS sample in dry THF (Fig. S2†). The ^11^B NMR spectra for both HPBS and LPBS showed peaks at 21.4 and 19.5 ppm. In addition, a smaller peak at 17.7 ppm was present exclusively in the LPBS spectrum. The appearance of peaks at similar chemical shifts suggests that boron is present in similar chemical environments in both HPBS and LPBS and maybe related to the appearance of mono-, di- and tri-borate esters, yet due to the polymer system being so susceptible to moisture we do not go into further detail here. Furthermore, the peak intensities in LPBS were 10-times larger than in HPBS, suggesting that a higher number of B(OSi)_3_ motifs are present in LPBS compared to HPBS, which is in line with the higher density of hydroxy end-groups present in LPDMS-OH and the previously discussed FTIR results.

The thermal stabilities of LPBS and HPBS were assessed using thermal gravimetric analysis (TGA) and compared to the respective PDMS-OH precursor polymers (Fig. 2). HPDMS-OH revealed a major degradation event at 439 °C where the former is tentatively assigned to the loss of low molar mass fragments and the latter to the thermal degradation of the polymer backbone. Upon reacting with boric acid to form HPBS, the onset of degradation only increases by a few degrees and the weight loss becomes more abrupt. The slight change in thermal stability fits well with the smaller amount of SiO–B observed in the FTIR results, indicative of the formation of fewer B(OSi)_3_ crosslinking sites. On the other hand, LPBS shows a much larger increase in the degradation temperature of ∼140 °C compared to its precursor PDMS-OH. The significant increase in thermal stability is in agreement with the earlier FTIR experiments indicating a much higher crosslinking density in LPBS than in HPBS.

**Fig. 3:**
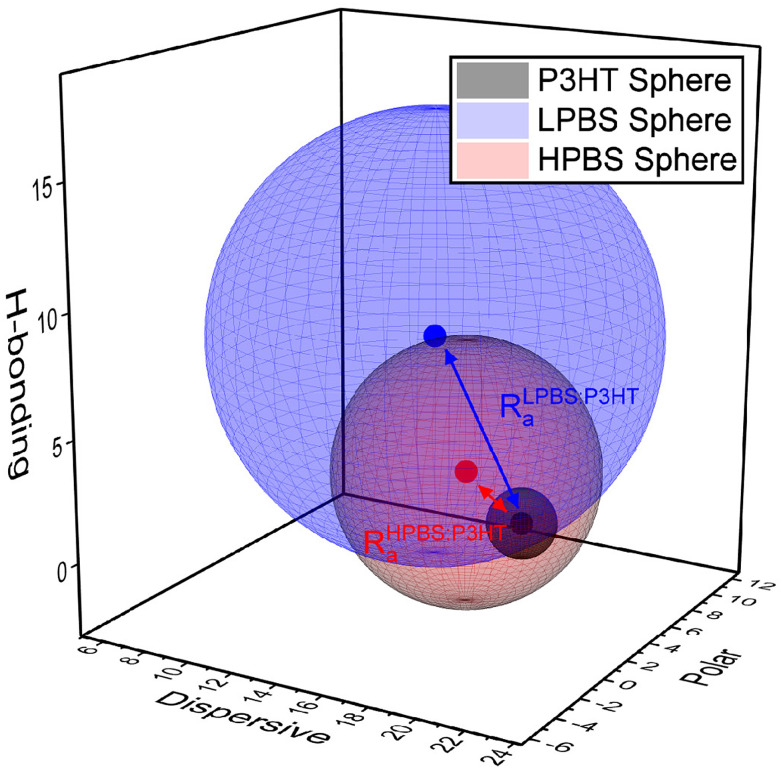
HSP co-ordinates of LPBS (blue), HPBS (red) and P3HT (black) and their associated spheres. The blue and red arrow highlights the *R*_a_ distance between LPBS and HPBS against P3HT, respectively.

To establish whether the different PBS samples may be miscible with the organic semiconductor P3HT, the Hansen Solubility Parameters (HSPs) of constituents were estimated. HSPs are used extensively for polymer processability to understand and optimise polymer blends to suit different applications. To start with, the dispersive (*δ*D), polar (*δ*P) and hydrogen bonding (*δ*H) parameters along with the sphere radius (*R*_0_) of each constituent need to be established to define its solubility space. In order to do this the solvent gradient method was used, wherein a ratio of ‘good’ and ‘bad’ solvents was used to create a library of solvent mixtures.^13^ Then, under controlled dissolving conditions, a 1 or 0 is attributed depending on whether the material dissolves or does not dissolve, respectively, in each solvent mixture. The resulting HSPs and radius are then solved by attempting to optimise the fit to 100% using the method laid out by Díaz de los Ríos *et al.*^31^ Chlorobenzene was chosen as the ‘good’ solvent and acetonitrile, methanol, hexane and triethylamine as the ‘bad’ solvents in ratios of good : bad of 5 : 0, 4 : 1, 3 : 2, 2 : 3, 1 : 4 and 0 : 5. The solubility results are shown in the ESI (Tables S3–S5†); however the *R*_0_ outcomes of P3HT and the two different polyborosiloxane elastomers are shown below (Fig. 3). The sphere created from the *R*_0_ value of P3HT is small (*R*_0_ = 1.4) signifying that it is insoluble in most solvents, as any solvent with an HSP value inside the sphere should dissolve the polymer. Organic semiconducting polymers, including P3HT, are notoriously insoluble in most non-aromatic and non-chlorinated organic solvents due to the planarity and the resulting strong π-stacking interactions of the polyaromatic backbone. On the other hand, LPBS and HPBS have larger spheres (*R*_0_ = 9.4 and 5.4, respectively) reflecting their higher solubility in numerous solvents. Furthermore, LPBS's sphere is much larger than HPBS's, due to a large contribution from *δ*H, suggesting that LPBS has strong hydrogen bonding interactions; however, in the context of this system the interactions are more likely to arise from lone pair donation from an oxygen to an empty p-orbital on the boron. To estimate the compatibility between P3HT and the PBS elastomer, the vector distance between two HSP values (*R*_a_) can also be calculated, where a smaller *R*_a_ indicates better miscibility between each of the constituents. As shown in Fig. 3, HPBS : P3HT has a much smaller *R*_a_ than LPBS : P3HT, suggesting that it is more miscible. The relative energy density (RED) can also be calculated *via* the ratio between the *R*_a_ value of the blend and the *R*_0_ value of the PBS elastomer where RED < 1 is an indication that the two materials in the blend are deemed miscible. For HPBS : P3HT, RED = 0.76 and for LPBS : P3HT, RED = 0.86 which show that P3HT may blend well with each elastomer. However, the lower RED value of HPBS : P3HT further indicates better miscibility than LPBS : P3HT.

**Fig. 4:**
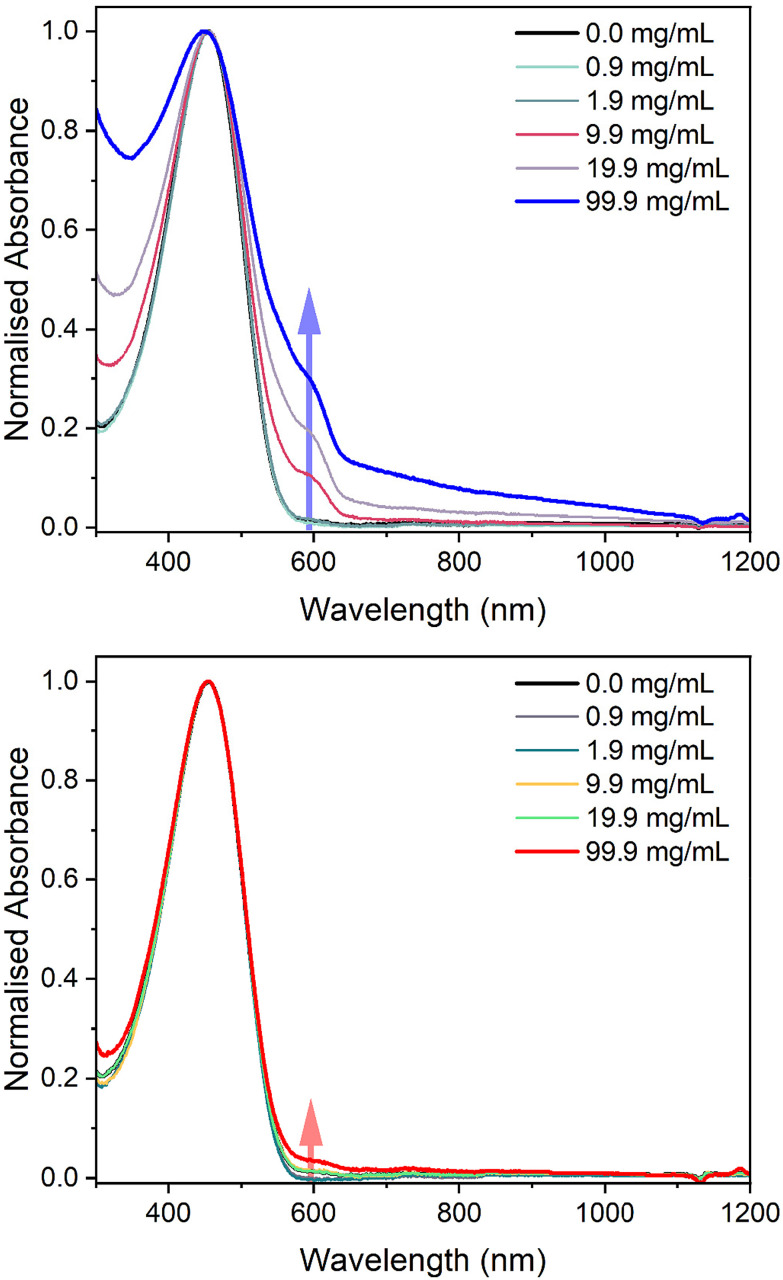
Normalised solution UV-vis absorbance spectra of LPBS : P3HT (top) and HPBS : P3HT (bottom) blends in chlorobenzene with an increase in the concentration of PBS with a constant concentration of 0.1 mg mL^−1^ P3HT. The spectra have been normalised to the S_0_ to S_1_ transition at 458 nm. The arrows highlight to the reader the P3HT aggregation peak at around 600 nm.

To initially investigate the miscibility between P3HT and the two elastomers, solution UV-vis absorbance spectroscopy was employed by dissolving the materials in chlorobenzene (Fig. 4). While keeping the concentration of P3HT in chlorobenzene constant at 100 μg mL^−1^, the amount of PBS was increased to observe any aggregation of P3HT, which would suggest immiscibility. The spectra of pure P3HT in chlorobenzene showed a single peak with a maximum absorbance at 458 nm. However, as the concentration of LPBS or HPBS was increased a new absorption band appeared at around 600 nm. The HSP experiments revealed that LPBS does not easily dissolve in chlorobenzene at room temperature, which subsequently results in an increase in background scattering with increasing LPBS concentration. In line with previous studies, the low energy shoulder at 600 nm is related to the P3HT aggregates formed in solution.^32^ The intensity of the shoulder for LPBS at 99.9 mg mL^−1^ is much higher than that of HPBS at the same concentration (0.29 *vs.* 0.04) which indicates that LPBS induces higher P3HT aggregation than HPBS. Agreeing with the lower RED value for HPBS : P3HT than LPBS : P3HT from the HSP experiments, the solution UV-vis absorbance results suggest a lower miscibility for P3HT with LPBS manifesting itself in P3HT aggregation.

The HSP and solution UV-vis absorbance results suggest that HPBS and P3HT are more compatible than LPBS and P3HT and therefore to assess the effects of compatibility the thermal and mechanical characteristics of the blends were investigated. To accomplish this, P3HT and PBS were blended in chlorobenzene with increasing P3HT concentration (0.1, 0.5, 1, 5 and 10 wt% in PBS). The complete methodology is detailed in the ESI,† but briefly, the two components were dissolved separately in chlorobenzene, combined, and stirred at 80 °C overnight. The chlorobenzene was removed slowly under reduced pressure by increasing the temperature from 20 °C to 120 °C. Upon examining the resulting solid, samples with more than 5 wt% of P3HT had formed inhomogeneous blends in which P3HT was visibly phase-separated from the elastomer (see Fig. S7 and S8†).

**Fig. 5:**
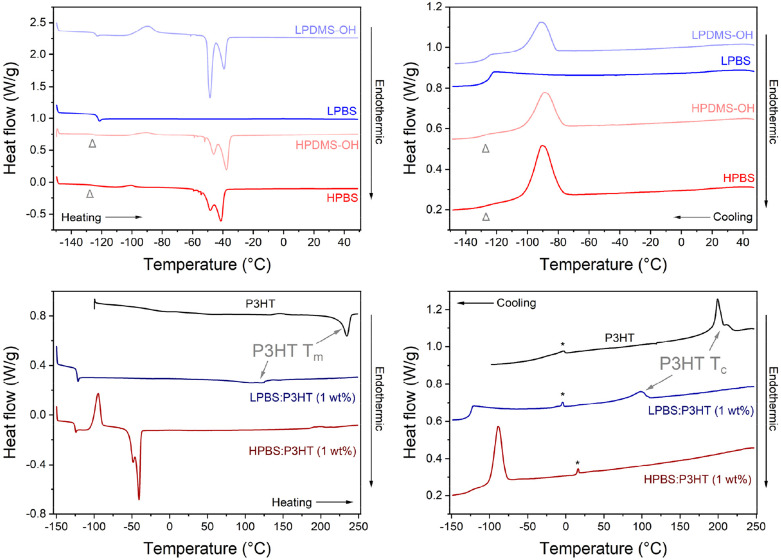
(Left column) Second heating and (right column) first cooling cycles of P3HT, PDMS-OH and PBS polymers, as well as PBS : P3HT blends recorded at a scan rate of 10 °C min^−1^ under a helium atmosphere. The light grey arrowhead indicates the *T*_g_ for HPDMS-OH and HPBS. The peaks marked with a star are measurement artefacts.

The thermal properties of the pure polymers, elastomers and the blends containing 1 wt% P3HT were measured using differential scanning calorimetry (DSC), using a heating rate of 10 °C min^−1^ under a helium atmosphere (Fig. 5, Tables S6 and S7†). Firstly, the thermal transitions of the precursor PDMS-OH polymers were compared to discern the effects of molar mass on thermal properties between LPDMS-OH and HPDMS-OH. In the cooling cycle for LPDMS-OH, an exothermic peak at −92 °C was assigned to polymer crystallisation (*T*_c_) with an associated enthalpy change (Δ*H*_c_) of 12.2 J g^−1^ and a step change at −126 °C due to a change in heat capacity at the glass transition temperature (*T*_g_). The same crystallisation event was observed for HPDMS-OH, albeit at a lower temperature (*T*_c_ = −72 °C; Δ*H*_c_ = 24.4 J g^−1^). The glass transition temperature (*T*_g_) was again observed around −125 °C; however, the associated step change was significantly less pronounced. In the heating cycle three distinct events were observed for both LPDMS-OH and HPDMS-OH: a glass transition (*T*_g_), an exothermic peak associated with cold crystallisation (*T*_cc_) and a bimodal endothermic peak as a result of polymer melting (*T*_m_). Whilst it has been observed that altering the molar mass of a polymer can affect the *T*_g_ in linear PDMS, the *T*_g_ for both PDMS-OH polymers was found to be identical (∼−125 °C).^33^ The appearance of *T*_cc_ at ∼−90 °C is related to experimental conditions, where upon cooling the polymer too quickly, not all crystallites have time to form before reaching *T*_g_. Upon heating again above *T*_g_, any nucleation sites will start to recrystallise. Assuming that all crystallites have formed at *T*_cc_, the total enthalpy of crystallisation (Δ*H*_c-total_) would be the sum of Δ*H*_c_ and Δ*H*_cc_, allowing for the crystalline fraction (*χ*_c_) to be estimated using an enthalpy change of 100% crystalline linear PDMS (Δ*H*_100%_ = 37.4 J g^−1^).^34^ The results conclusively show that HPDMS-OH has a higher crystalline fraction than LPDMS-OH (0.73 *vs.* 0.59) assigned to the much higher molar mass. At higher temperatures, the crystallites in both PDMS-OH polymers start to melt as evidenced by the bimodal endothermic peak (*T*_m_). The bimodal melt is routinely observed for PDMS and has been suggested to be related to the melt of different metastable crystallites.^35^ The *T*_m_ used here is the average between the two peaks and for LPDMS-OH and HPDMS-OH the values are −49 and −38 °C. It is also worth noting here that the two peaks (about −50 and −40 °C) change in heat flow intensity between the two PDMS-OH polymers, believed to be the result of different molar masses.^35^ LPDMS-OH exhibited a lower *T*_m_ than HPDMS-OH, yet Δ*H*_m_ for LPDMS-OH is more than twice its Δ*H*_c-total_, whereas Δ*H*_m_ for HPDMS-OH does not significantly change from its Δ*H*_c-total_. The increase in Δ*H*_m_ for LPDMS-OH compared to Δ*H*_c-total_ is indicative of an underlying supramolecular structure, previously assigned to hydrogen bonding interactions between the hydroxy end-groups.^33^ While the two PDMS-OH polymers exhibited comparable thermal properties, the PBS elastomers, conversely, exhibited stark differences between the lower and higher molar mass samples. The thermogram of LPBS is completely different from the LPDMS-OH thermogram, where all transitions apart from *T*_g_ are no longer observable. The loss of most thermal transitions in the LPBS is further evidence for the formation of a crosslinked network lacking significant crystalline structure. In contrast, HPBS revealed very similar thermal transitions to HPDMS-OH with the only difference being a small decrease in Δ*H*_cc_ (1.0 *vs.* 3.2 J g^−1^), resulting in a smaller *χ*_c_ (0.68 *vs.* 0.73). The resemblance observed between the HPDMS-OH and HPBS thermograms strongly suggests that HPBS has preserved a nearly identical microstructure, likely attributed to the limited number of crosslinking sites.

As a result of the differences in polymer networks between LPBS and HPBS, the thermal transitions upon blending 1 wt% P3HT into each elastomer are very different, in both the heating and cooling cycles (Fig. 5). P3HT showed *T*_m_ and *T*_c_ peak maxima at 235 and 199 °C with an associated Δ*H*_m_ of 14.9 J g^−1^ and Δ*H*_c_ of 14.3 J g^−1^, respectively. Introducing 1 wt% of P3HT into HPBS shifted the *T*_m_, *T*_c_ and *T*_cc_ of HPBS by ∼5 °C to higher temperatures, while Δ*H*_m_ remained unchanged, Δ*H*_c_ of the HPBS : P3HT blend decreased (14.3 J g^−1^) and Δ*H*_cc_ increased (16.5 J g^−1^). The appearance of a *T*_cc_ in the thermogram, as well as the alteration in enthalpy change between the pure elastomer and the blend, indicates that the addition of P3HT has a profound effect on the crystallisation kinetics of the HPBS : P3HT blend.

The absence of a defined crystallisation peak for P3HT provides evidence that P3HT has blended well with HPBS. This is in good agreement with the solution UV-vis absorbance results where an increasing HPBS content did not induce any strong P3HT aggregation yet it is conflicting with the images of the blends (Fig. S7 and S8†). We therefore suggest that HPBS and P3HT are blending well on the microscale; however, on the macroscale, relatively large P3HT rich domains are phase separating. Contrary to the P3HT : LPBS blend where the introduction of 1 wt% P3HT into LPBS led to the presence of a new endothermic and exothermic peak in the respective heating and cooling cycles. The peaks at 120 °C and 98 °C are assigned to the *T*_m_ and *T*_c_ of P3HT crystallites within the blend. Their appearance suggests that P3HT has not blended well with the LPBS, which again is in agreement with the solution UV-vis absorbance experiments and the increased RED value obtained from the HSPs.

The mechanical properties of the pure PBS elastomers and blends were assessed by rheology using 20 mm parallel plates at 25 °C. Strain amplitude experiments on the pure PBS samples were initially carried out to assess the critical strain (Fig. S10†). This was done in order to ensure that the oscillation sweep measurements on the blends were within the linear viscoelastic region (LVR). The LVR is the range in which measurements can be carried out without destroying the structure of the sample and the critical strain signifies this maximum strain.^36^ At a constant frequency of 1 Hz against increasing strain, the elastic modulus (*G*′) of LPBS deviates from the plateau at a strain of ∼25%. HPBS, however, starts to show a decrease in *G*′ at a strain in excess of ∼40%. The absolute value of the critical strain was then taken to be 24 and 41% for LPBS and HPBS, respectively, with a standard deviation of ±5% in *G*′.^36^ The *G*′ of LPBS within the LVR was ∼125 kPa, whereas HPBS showed a much lower *G*′ within the LVR of only ∼21 kPa. The differences in critical strain and *G*′ between the two PBS samples are inferred due to the number of chain end crosslinks, agreeing with the increase in the intensity of the SiO–B stretch for LPBS from the FTIR measurements. The small amplitude oscillatory shear (SAOS) experiments across all samples were performed at a constant strain of 10% assumed to be well within the LVR of the different PBS samples.

**Fig. 6:**
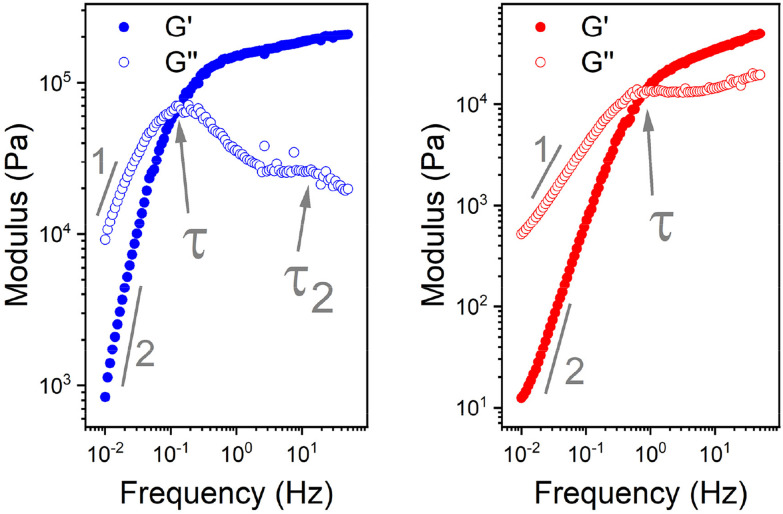
Double log plot of the modulus as a function of frequency of LPBS (left) and HPBS (right), showing the elastic modulus *G*′ (closed circles) and viscous modulus *G*′′ (open circles) obtained from SAOS rheology experiments at a constant strain of 10% between 20 mm parallel plates at 25 °C. The arrows indicate the crossover point where *τ* and *τ*_2_ are obtained from. The lines also indicate the gradient at the terminal region associated with Maxwell equations of frequency for a viscoelastomeric material.

The *G*′ and *G*′′ of the pure PBS, PDMS-OH precursors, and blends with an increase in angular frequency (0.01 to 50 Hz) and constant strain (10%) were extracted from the SAOS experiments (Fig. 6, Fig. S11, S12 and S14†). When comparing the SAOS results for PDMS-OH and PBS samples, PDMS-OH shows a linear increase in *G*′ and *G*′′ with respect to increasing frequency whereas PBS shows a crossover of *G*′ and *G*′′. Previous literature reports on PBS rheology showed that PBS acts as a liquid at low oscillating frequencies, whereas adopting more solid-like properties at higher frequencies above the crossover point.^23,28,29,37,38^ According to the Maxwell model, and at a constant temperature, *G*′ and *G*′′ can be expressed as eqn (1) and (2)1
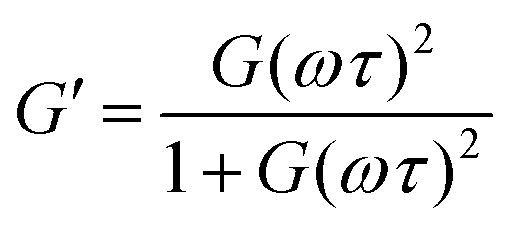
2
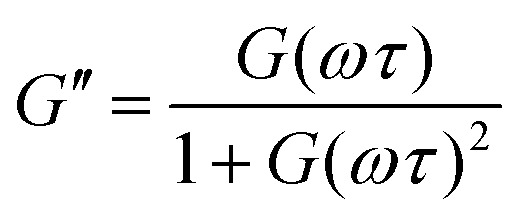
where *ω* is the angular frequency, *τ* is the relaxation time and *G* is the modulus.^36^ At very low frequencies (*ω* ≪ 1/*τ*), in the terminal regions, eqn (1) and (2) can be reduced to

3 *G*′ = *G*(*ωτ*)^2^

4 *G*′′ = *G*(*ωτ*)

In the terminal region *G*′ and *G*′′ linearly increase with frequency with gradients of 2 and 1, respectively, for an ideal viscoelastic material according to the Maxwell model. Increasing the frequency of oscillation led to a crossover point between *G*′ and *G*′′, signifying the change between its liquid state and solid state, and the reciprocal of this frequency is the terminal relaxation time of the material (*τ*). At the crossover point for LPBS and HPBS *τ* is calculated to be 1.1 s and 0.2 s. The stark difference in *τ* between the two elastomers clearly shows the influence of molar mass and/or crosslinking density on the relaxation dynamics of the resulting elastomer. This very phenomenon was explored by Seetapan *et al.*, where they found that synthesising PBS from PDMS-OH with *M*_n_ above and below the *M*_c_ of linear PDMS led to a stark difference in *G*′ and *τ*.^39^ They theorised and calculated that synthesising PBS from PDMS-OH with *M*_n_ > *M*_c_ that trapped entanglements contributed to the relaxation, whereas using *M*_n_ < *M*_c_ it did not. Yet, when using PBS synthesised from PDMS-OH with *M*_n_ < *M*_c_ they observed a much larger *G*′, which they attributed to a higher SiO–B crosslinking density. According to the FTIR spectroscopy results, LPBS contains a higher number of SiO–B stretches compared to HPBS and the *M*_n_ values of LPDMS-OH and HPDMS-OH measured using SEC are below and above the respective *M*_c_ values of linear PDMS. The data shown here fits well with the theory from Seetapan *et al.* indicating that the higher *G*′ within the LVR and *τ* exhibited by LPBS compared to HPBS arises primarily from the higher crosslinking density, whereas both entanglement and crosslinking sites contribute to the *G*′ of HPBS. The relaxation process at the crosslinking sites is assigned to a dynamic covalent bonding mechanism as discussed by Kurkin *et al.*^19^ At higher frequencies, a relaxation process is observed in LPBS with a maximum *G*′′ value at ∼14 Hz, resulting in a secondary relaxation time (*τ*_2_) of 12 ms. The rather low relaxation time has been proposed to be the result of the formation of locally branched networks arising from the formation of dynamic dative bonds between oxygen and boron at the borate crosslinking sites or hydrogen bonding between unreacted OH groups.^29^ The concept is based on the dissociation and re-association of transient networks in addition to physical B–O–Si crosslinking according to the “sticky reptation” model.^40,41^ A linear fit of the LPDMS-OH SOAS results also shows that *G*′ and *G*′′ also cross over at a similar time scale of ∼11 ms suggesting that the transient network in LPDMS-OH is also present in LPBS. On the other hand, HPBS exhibits a lower number of crosslinking sites, but is synthesised from a PDMS-OH precursor above the *M*_c_ of linear PDMS. Therefore, the relaxation mechanism of HPBS can be assumed to arise due to polymer entanglements and dynamic covalent bonding. In summary, the different relaxation times observed between LPBS and HPBS are the result of a complex interplay between the number of dynamic and physical crosslinks, in addition to different entanglements caused by the different PDMS-OH precursor polymers employed for the synthesis of the respective PBS elastomers.

**Fig. 7:**
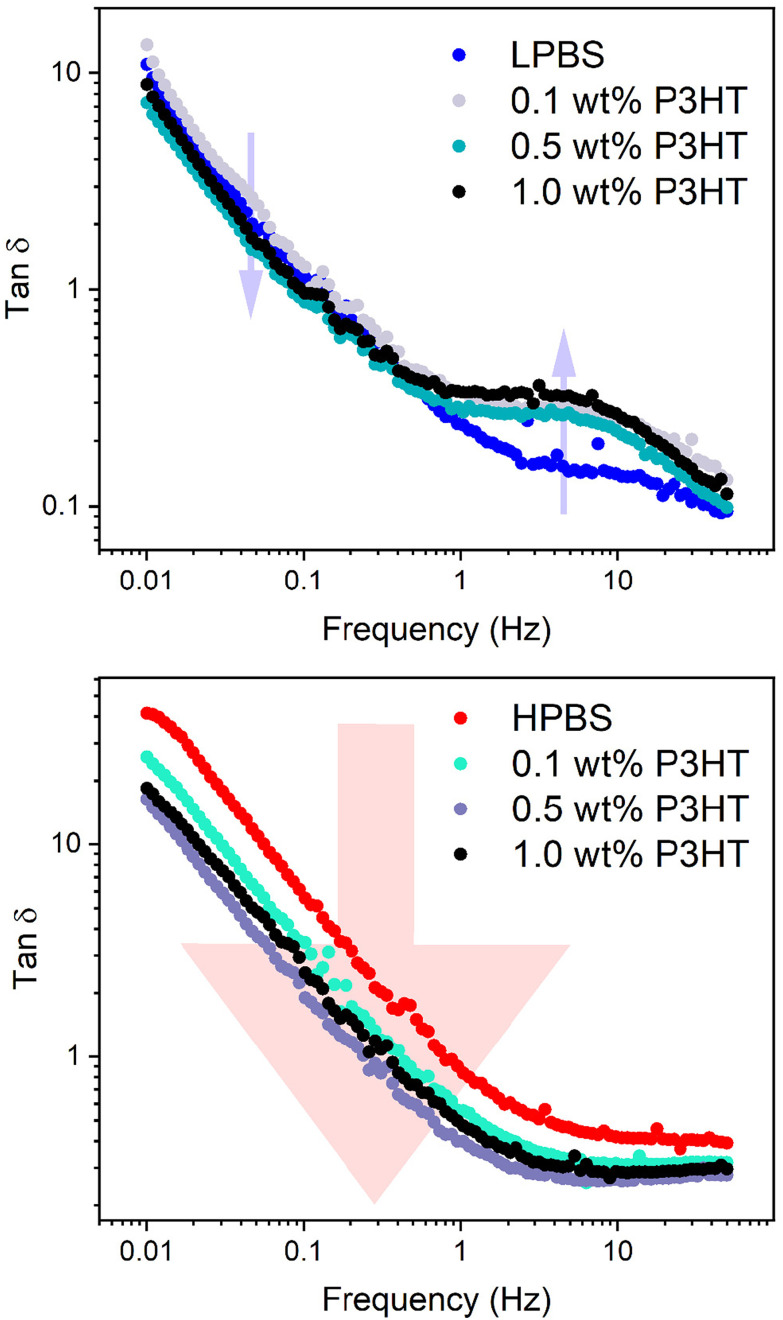
Double log plot of tan *δ vs.* frequency for LPBS : P3HT (top) and HPBS : P3HT blends (bottom) extracted from the SAOS experiments. The arrow on the plot containing the data from the LPBS : P3HT samples indicates to the reader where the change in tan *δ* arises with an increase in the P3HT content, located at *τ*_2_. The arrow on the plot containing the data from the HPBS : P3HT samples indicates to the reader how tan *δ* decreases across the entire frequency range with an increase in the P3HT content.

SAOS experiments on the PBS : P3HT blends were then carried out under the same conditions (10% strain) to assess the effects of increasing the P3HT content on the mechanical dynamics (Fig. S12, S14, Tables S8 and S9†). It should also be noted here that the batch of P3HT used is assumed to be above the *M*_c_ of P3HT according to the experiments carried out by Koch *et al.*^42^ Upon adding 0.1 wt% P3HT to LPBS, *τ* does not significantly change from pure LPBS (*τ* = 1.2 s) and the gradient in the terminal region remains the same. The same effect is seen upon adding 0.1 wt% P3HT to HPBS, suggesting that the very small amount of P3HT does not disrupt the relaxation network. However, increasing the P3HT content in LPBS to 1 wt% almost doubles the relaxation time (*τ* = 1.9 and 1.6 s for 0.5 and 1.0 wt%, respectively) and the gradient in the terminal region deviates from the Maxwell model. Again a similar effect is observed in the HPBS : P3HT blends where increasing the P3HT content led to a maximum *τ* of 0.6 s at 0.5 wt%. The deviation from the Maxwell model in the terminal region for both LPBS and HPBS blends with P3HT is ascribed to the incorporation of a polymer that is not viscoelastic in the measured frequency range. The increase in *τ* with respect to P3HT in the PBS samples suggests that there is a critical point between 0.1 and 0.5 wt% of P3HT that causes a dramatic change in the dynamic network, preventing the relaxation processes discussed earlier for LPBS and HPBS. Similarly, the secondary relaxation *τ*_2_ for the LPBS samples increases from 12 to 24 ms upon adding 1 wt% P3HT. The effect is easier to recognise from the double log plot of tan *δ vs.* frequency, where tan *δ* is *G*′′/*G*′ between 1 and 50 Hz (Fig. 7, S13 and S15†). Tan *δ* increases for all LPBS samples containing P3HT at the secondary relaxation point; however, in the terminal region (below ∼1 Hz), tan *δ* decreases due to an increase in *G*′. This interesting rheological result shows that the incorporation of P3HT into LPBS causes a dampening effect on the transient network, perhaps preventing hydrogen/dative bonding between LPBS chain ends. Conversely, tan *δ* decreases over the measured frequency range for HPBS : P3HT blends, indicating a harder material with increasing P3HT concentration. In summary, adding 1 wt% P3HT clearly disrupts the network for both LPBS and HPBS as seen by the increase in *τ*; however, the double log plot of tan *δ vs.* frequency highlights the intricate mechanisms affecting the two viscoelastic networks and further work is needed to gain a deeper understanding of the disruptions caused by P3HT in the PBS networks.

## Conclusions

With the intention to obtain a homogeneous blend between a semiconducting polymer (P3HT) and an elastomer (PBS) exhibiting supramolecular interactions to exploit in flexible electronics, it was found that miscibility played a crucial role. The stark differences between using a polymer of molar mass above and below the *M*_c_ of linear PDMS to create a polyborosiloxane led to elastomers with different thermal and mechanical properties. LPBS showed a complete loss in observed crystallisation and melting transitions in the precursor LPDMS-OH, and, according to the SAOS experiments in the low frequency terminal region, obeyed the Maxwell model well. This suggests that LPBS is a well-defined viscoelastomer whereby its relaxation mechanism is dominated by dynamic crosslinking. On the other hand, HPBS retained both crystallisation and melting transitions, with a similar *χ*_c_ to HPDMS-OH, indicating that the smaller number of SiO–B crosslinks observed by FTIR does not affect the polymers’ crystalline structure. SAOS experiments on HPBS revealed much quicker relaxation than for LPBS, which we assigned to polymer entanglements having a major contribution rather than the dynamic crosslinking mechanism observed in LBPS. Alongside this, HPBS does not fit the Maxwell model, indicating that the material deviates from a true viscoelastic response to stress.

The HSP results suggested that P3HT and PBS elastomers are deemed miscible, yet upon blending P3HT into the PBS elastomers, it became apparent that phase separation was unavoidable when blending more than 1 wt% P3HT in PBS. From the DSC measurements, however, 1 wt% P3HT in HPBS blended well in the microstructure, made apparent by no appearance of the *T*_c_ of P3HT, which indicated that no P3HT crystallite phases were present. On the other hand, phase separation between P3HT in LPBS led to a clear *T*_c_ in the DSC due to the lower miscibility between the two components. The mechanical properties of each elastomer were not drastically altered upon introducing 0.1 wt% P3HT as seen by a similar *τ*; however, >0.5 wt% P3HT, the blends showed a longer *τ*. Also, the double log plot of tan *δ vs.* frequency showed that the increase in P3HT content disrupted the supramolecular structure of LPBS and HPBS differently.

The study underscores that blending a conjugated polymer with a viscoelastomer is more complex than initially anticipated. The delicate supramolecular interactions responsible for the viscoelastomer's mechanical properties can be disrupted even by incorporating small amounts of conjugated polymer. Additionally, the study highlights the role of molar mass in polymeric networks, showing that significantly different viscoelastomers can be produced simply by altering the molar mass of the precursor polymer. As a result, factors such as molar mass, physical and supramolecular crosslinking, and miscibility must be carefully considered when designing conjugated polymer-elastomer blends for flexible electronics.

## Data availability

Data for this article, including DSC, TGA, FTIR, NMR, rheology, SEC, and UV-vis spectra, are available at UCL Research Data Repository at https://doi.org/10.5522/04/26371144.v1.

## Conflicts of interest

There are no conflicts to declare.

## Supplementary Material

LP-002-D4LP00163J-s001
