# Supplementary material for: Will it blend? Exploring the viscoelastic characteristics of P3HT-polyborosiloxane blends towards flexible electronic materials
Source: RSC Appl Polym. 2024 Oct 10;2(6):1182–92. doi: 10.1039/d4lp00163j (PMC11498086; doi:10.1039/d4lp00163j)
Supplement: LP-002-D4LP00163J-s001 [file LP-002-D4LP00163J-s001.pdf]

Supplementary Information (SI) for RSC Applied Polymers.
This journal is © The Royal Society of Chemistry 2024

Supporting Information

Will it blend? Exploring the viscoelastic characteristics of P3HT-polyborosiloxane blends towards flexible electronic materials

Peter A. Gilhooly-Finn[a]*, Megan M. Westwood[a,b] and Bob C. Schroeder[a]*

[a]Department of Chemistry, 20 Gordon Street, London, WC1H 0AJ, United Kingdom.
[b]Department of Chemistry and Chemical Engineering, Chalmers University of Technology, 41296 Göteborg, Sweden.

*Corresponding Authors: p.finn@ucl.ac.uk; b.c.schroeder@ucl.ac.uk

Table of Contents

1. Materials 2
2. Synthesis of polyborosiloxane 2
3. PBS:P3HT blending procedure 2
4. Nuclear Magnetic Resonance (NMR) 3
5. Size Exclusion Chromatography (SEC) 4
6. Fourier Transform Infra-red (FTIR) and UV-vis spectroscopy 5
7. Photographs of LPBS:P3HT and HPBS:P3HT blends 6
8. Thermogravimetric analysis (TGA) 6
9. Hansen Solubility Parameter (HSP) Study 7
10. Differential Scanning Calorimetry (DSC) 9
11. Rheology 9
12. References 15

1. Materials

Poly(3-hexylthiophene) was synthesised according to previous literature.[1] The regioregularity was estimated to be 95 % via ^{1}H NMR and the number-average molar mass (M_n) and dispersity (Đ), determined via gel permeation chromatography in chlorobenzene at 80 °C, are 41 kg mol^{-1} and 1.7 respectively. The low and high molar mass hydroxy-terminated polydimethylsiloxanes (LPDMS-OH and HPDMS-OH) were purchased from Alfa Aesar and Sigma-Aldrich. Boric acid ($B(OH)_3$) (>99 % purity) was purchased from Sigma-Aldrich and dried at 120 °C under vacuum before use. All solvents were purchased from Honeywell or Fisher. All chemicals were used as received unless otherwise stated.

2. Synthesis of polyborosiloxane

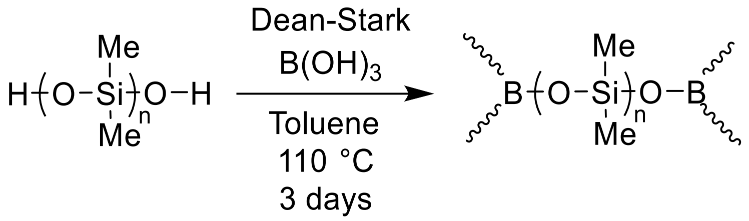

Scheme S1. *Synthetic route to produce PBS from PDMS-OH.*

The general synthesis of polyborosiloxane was adapted from the literature.[2] PDMS-OH (20 g, 270.3 mmol) was dissolved in toluene (250 mL) in a two neck round bottom flask attached with a Dean-Stark apparatus, condenser and internal thermometer. Boric acid was added (2 g, 32.3 mmol) and the suspension was stirred vigorously for 10 mins at room temperature to ensure it is fully mixed. Then the reaction mixture was heated to reflux, and the temperature was monitored between 110-115 °C for 3 days. During this time the suspension persisted. After cooling to room temperature, the toluene was removed under reduced pressure and the sample was further dried in a vacuum oven at 40 °C overnight to give the crude product as a cloudy viscous liquid. The crude was redissolved in hexanes (~350 mL) using a combination of stirring, heating, and sonicating, and then filtered through a 0.45 µm PTFE syringe filter to remove any excess and undissolved boric acid. The hexane was removed under reduced pressure and then dried in the vac oven at 120 °C overnight to give PBS as a clear solid. The PBS was stored under vacuum to avoid moisture accumulation in the sample. Prior to any measurements the samples were heated to 120 °C under vacuum to remove any residual moisture.

3. PBS:P3HT blending procedure

PBS (250 mg mL^{-1}) and P3HT (25 mg mL^{-1}) were separately dissolved in chlorobenzene at 80 °C for 3 hrs and then combined in ratios of PBS:P3HT 900:100, 950:50, 990:10, 995:5 and 999:1 (w/w). The solutions were stirred overnight at 80 °C to ensure full mixing. After the magnetic stirrer bars were removed, they were placed in a vacuum oven at room temperature for 4 hrs, then 70 °C for 2 hrs, then 120 °C overnight to dry. The importance of slowly raising the temperature under vacuum is to prevent trapped air and residual solvent from rapidly escaping and spilling the sample. The PBS:P3HT blends were then transferred to clean vials and stored under vacuum to avoid moisture accumulation in the sample. Prior to any measurements the samples were heated to 120 °C under vacuum to remove any residual moisture.

4. Nuclear Magnetic Resonance (NMR)

Proton NMR of P3HT was carried out on a Bruker Avance III 400 in $CDCl_3$ (Thermo Scientific) at room temperature. The regioregularity of P3HT was estimated from the ratio between the peaks between 2.5 and 2.8 ppm. Boron NMR of LPBS and HPBS was carried out on a Bruker Avance NEO 700 with the samples dissolved in dry THF in oven dried vials. Approximately 30 mg of each PBS sample was dissolved in 0.7 mL of dry THF. After dissolution a white precipitate appeared (assumed to be boric acid) and then everything was transfer to an NMR tube. The spectra were baselined corrected manually using a polynomial fit.

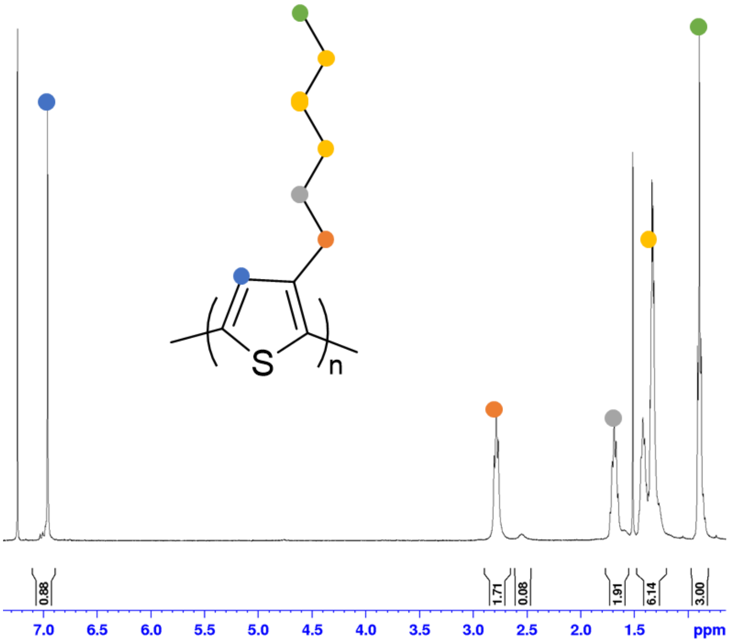

Figure S1. *^{1}H NMR spectra of the P3HT used in this study carried out on a 400 MHz in $CDCl_3$. The different colours spots on the peaks and chemical structure represent where the peaks arise from in the spectrum.*

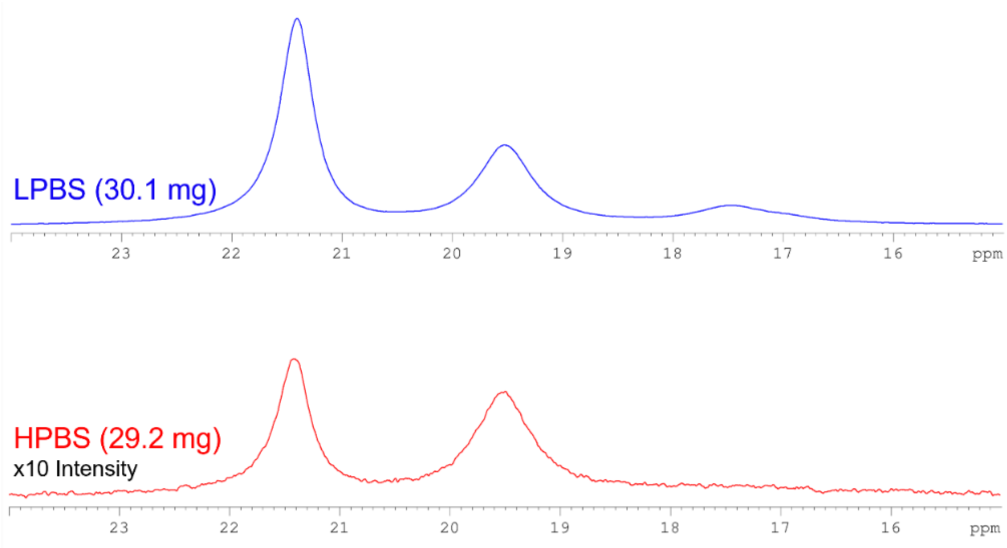

Figure S2. *^{11}B NMR of LPBS and HPBS dissolved in dry THF carried out on a 700 MHz NMR.*

5. Size Exclusion Chromatography (SEC)

SEC was carried out on a Shimadzu LC-2030c system (Controller, Pump, Autosampler and Oven) with an attached refractive index detector RID-20A. The mobile phase was run through 1 and 2 Agilent PLgel 10 µm MIXED-B 300 x 7.5 mm columns as the stationary phase for PDMS-OH and P3HT samples respectively. The guard column attached in line with the analytical column was an Agilent PLgel 5 µm MIXED guard 50 x 7.5 mm. The system was calibrated to known polystyrene standards between 580 - 7,500,000 g mol^{-1} (EASICAL PS-1) purchased from Agilent Technologies. The polymers were run using chlorobenzene (HPLC grade, Honeywell) as the mobile phase with the column housed in an oven at 80 °C. All samples were made to 1 mg mL^{-1} in chlorobenzene and dissolved thoroughly using either heat or sonication. Then they were left at room temperature overnight before filtering through a 13 mm (Ø) 0.45 µm PTFE syringe filter before injecting into the SEC.

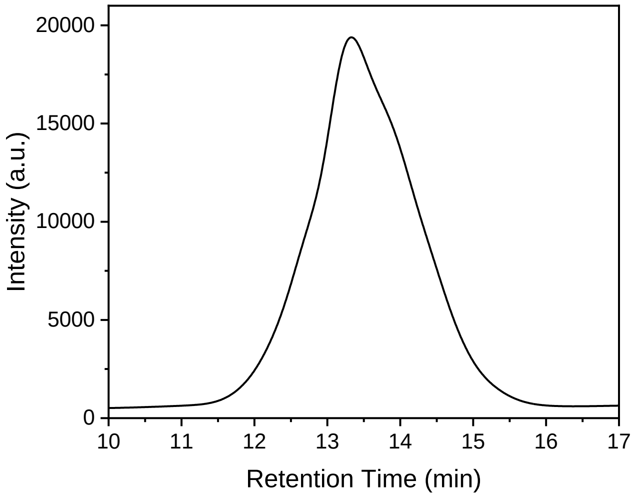

Figure S3. *SEC trace of the P3HT batch used in this study, run in chlorobenzene at 80 °C through 2 columns.*

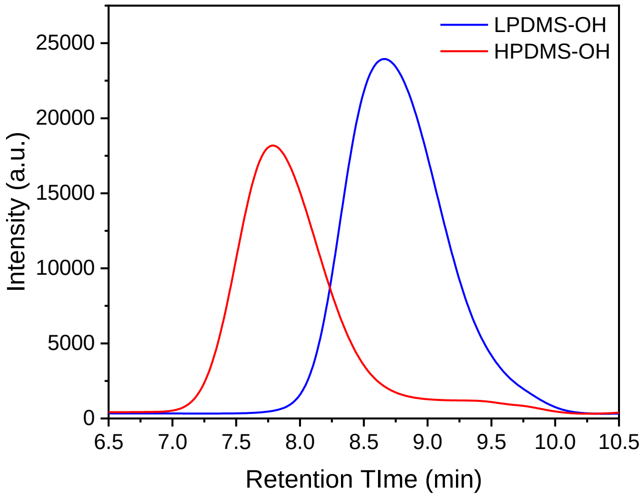

Figure S4. *SEC trace of HPDMS-OH (red) and LPDMS-OH (blue) used in this study, run in chlorobenzene at 80 °C though 1 column.*

__Table S1.__ Table showing the molar mass calculated from the SEC measurements.

**Polymer**	**M_n (kg mol^{-1})**	**M_w (kg mol^{-1})**	**Đ**
P3HT	57.5	115.5	2.0
LPDMS-OH	4.6	9.7	2.1
HPDMS-OH	40.0	85.0	2.1

6. Fourier Transform Infra-red (FTIR) and UV-vis spectroscopy

FTIR spectroscopy was carried out on a Bruker Platinum ATR in reflection mode. The transmission was recorded and converted to absorbance. Solution UV-vis spectroscopy was carried out on a Shimadzu UV-3600i Plus in 1 mm path length quartz cuvettes. The concentration of P3HT in chlorobenzene (Honeywell) was kept constant at 0.1 mg mL^{-1} whereas PBS was increased in concentration from 0 to 99.9 mg mL^{-1} and the total concentration ranges from 0.1 to 100 mg mL^{-1} according to the table below. Each spectrum was baselined to blank chlorobenzene.

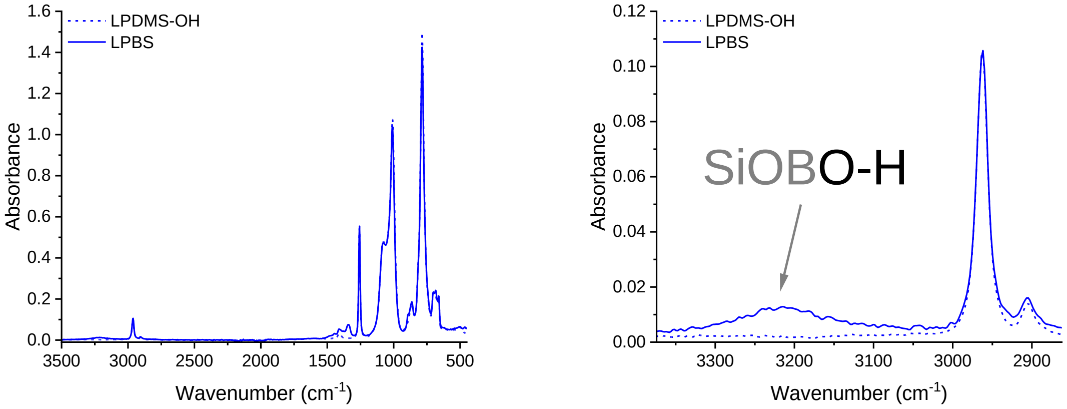

***Figure S5.** FTIR spectrum of LPDMS-OH (light blue) and LPBS (blue) (left). Zoomed in section of where the O-H stretches appear (right).*

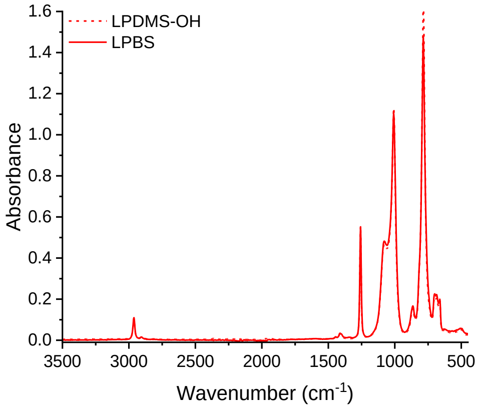

***Figure S6.** FTIR spectrum of HPDMS-OH (light red) and HPBS (red).*

Table S2. *Table showing the concentrations used in the solution UV-vis absorbance spectrums.*

**Sample Name (Legend on plot)**	**Concentration (mg mL^{-1})**		
	P3HT	*PBS*	*Total*
0.0 mg mL^{-1}	0.1	0.0	0.1
0.9 mg mL^{-1}	0.1	0.9	1
1.9 mg mL^{-1}	0.1	1.9	2
9.9 mg mL^{-1}	0.1	9.9	10
19.9 mg mL^{-1}	0.1	19.9	20
99.9 mg mL^{-1}	0.1	99.9	100

7. Photographs of LPBS:P3HT and HPBS:P3HT blends

For the images ~20 mg of each polymer blend was squashed between two microscope slides, placed on a light and piece of white paper, and the images were captured using a mobile phone camera.

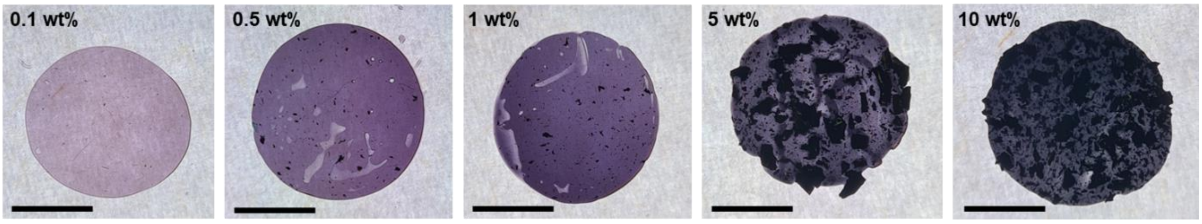

Figure S7. *Images of LPBS:P3HT blends with increasing P3HT content from 0.1 to 10 wt% (scale bar = 1 cm).*

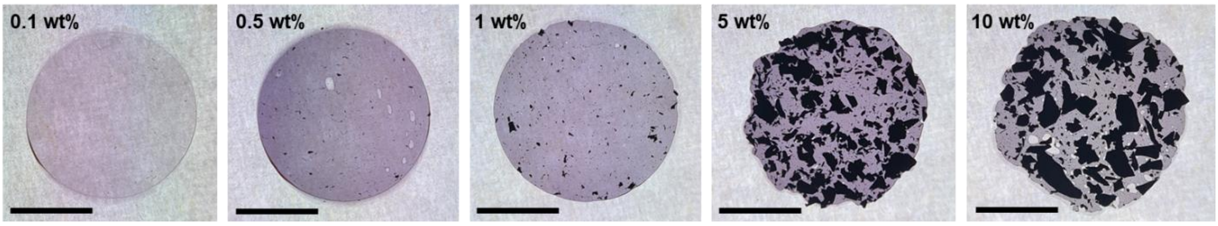

Figure S8. *Images of HPBS:P3HT blends with increasing P3HT content from 0.1 to 10 wt% (scale bar = 1 cm)*

8. Thermogravimetric analysis (TGA)

TGA was carried out on a TA Instruments TGA 5500 by weighing 5 mg of each sample into a platinum pan, heating to 600 °C from 45 °C at a rate of 10 °C min^{-1} under a nitrogen atmosphere and measuring the weight loss. The onset of degradation was obtained from the intersection of two tangents. It is noteworthy to mention that HPDMS-OH has two degradation events, a shallow one at 217 °C and main one at 439 °C. We hypothesise that the low temperature weight loss is caused by the loss of residual solvent or low molecular weight impurities in the commercial sample.

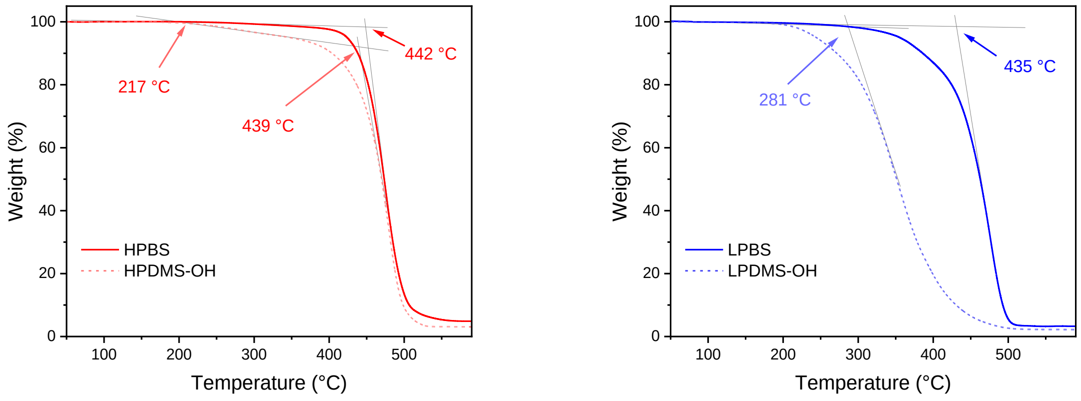

Figure S9. *TGA thermograms of HPBS (red) and HPDMS-OH (light red) (left) and LPDMS-OH (light blue) and LPBS (blue) (right). Where the grey lines overlap is where an onset of degradation was taken from.*

9. Hansen Solubility Parameter (HSP) Study

The total solubility parameter δ can be split up into its dispersive (δD), polar (δP) and hydrogen bonding (δH) contributions through the relation,

$$\delta^2 = \delta D^2 + \delta P^2 + \delta H^2$$

Alongside this, the hypothetical Hansen sphere (R_0) of each material is the space where 'good' solvents are enclosed, and 'bad' solvents are outside. The distance (R_a) between two constituents 1 and 2 can also be estimated using the following equation,

$$R_a^2 = 4(\delta D_1 - \delta D_2)^2 + (\delta P_1 - \delta P_2)^2 + (\delta H_1 - \delta H_2)^2$$

which denotes how close they are in space. The smaller R_a is between 1 and 2 the more miscible they are deemed. The relative energy density (RED) is the ratio between R_a/R_0 and if RED < 1 then they are can also be deemed miscible.

The HSP of P3HT, HPBS and LPBS were determined using the solvent gradient method adapted from the literature.[3] Solvents mixtures of 1 'good' (chlorobenzene) and 4 'bad' (methanol, acetonitrile, hexane, triethylamine) solvents in ratios of good:bad = 0:5, 1:4, 2:3, 3:2, 4:1 and 5:0 (v/v) were used to dissolve each material. A 1 mg mL^{-1} solution was made up using each solvent mixture for each constituent and then sonicated for 30 minutes in a Limplus Ultrasonic Cleaner LS-5D at room temperature and then left overnight. If the sample had fully dissolved, then it was given a '1' and if it had not it was given a '0'. The HSP each P3HT, HPBS and LPBS were solved using the evolutionary solver in Microsoft Excel following the method outlined by Díaz de los Ríos *et al.*[4]

Table S3. *HSP parameters of the solvents and mixtures used in this study.*

Solvents	δD	δP	δH
Chlorobenzene (CB)	19	4.3	2
CB:MeOH (80:20)	18.14	5.9	6.06
CB:MeOH (60:40)	17.28	7.5	10.12
CB:MeOH (40:60)	16.42	9.1	14.18
CB:MeOH (20:80)	15.56	10.7	18.24
Methanol (MeOH)	14.7	12.3	22.3
CB:AcN (80:20)	18.26	7.04	2.82
CB:AcN (60:40)	17.52	9.78	3.64
CB:AcN (40:60)	16.78	12.52	4.46
CB:AcN (20:80)	16.04	15.26	5.28
Acetonitrile (AcN)	15.3	18	6.1
CB:TEA (80:20)	18.3	3.52	1.8
CB:TEA (60:40)	17.6	2.74	1.6
CB:TEA (40:60)	16.9	1.96	1.4
CB:TEA (20:80)	16.2	1.18	1.2
Triethyl amine (TEA)	15.5	0.4	1
CB:Hex (80:20)	18.18	3.44	1.6
CB:Hex (60:40)	17.36	2.58	1.2
CB:Hex (40:60)	16.54	1.72	0.8
CB:Hex (20:80)	15.72	0.86	0.4
Hexane (Hex)	14.9	0	0

Table S4. *HSP values estimated from the Evolutionary solver for each material used in this study.*

Sample	δD	δP	δH	R_0	Fit%
P3HT	18.8	3.7	1.0	1.4	99
LPBS	14.4	4.5	7.8	9.1	97
HPBS	16.3	3.7	2.7	5.4	100

Table S5. *The R_a and RED values of Blending P3HT with either HPBS or LPBS.*

Blend	R_a	RED
P3HT:HPBS	7.1	0.76
P3HT:LPBS	11.1	0.82

10. Differential Scanning Calorimetry (DSC)

DSC was carried out on a TA DSC2500 with TA LN2P liquid nitrogen pump attached by cycling between -150 and 250 °C under a helium atmosphere. Between 5-8 mg of each material was weighed out on a 6 d.p. balance (Sartorius Quintix 35-15) into hermetically sealed TA Tzero aluminium pans. The values in table S6 are taken from the second cooling and second heating scan.

Table S6. *Values for the thermal transitions extracted from the DSC traces.*

Sample	T_g (°C)	T_m (°C) [ΔH_m (J g^{-1})]	T_c (°C) [ΔH_c (J g^{-1})]	T_{cc} (°C) [ΔH_{cc} (J g^{-1})]
LPDMS-OH	-124	-49 [34.3]	-92 [12.2]	-90 [9.9]
HPDMS-OH	-125	-38 [25.2]	-78 [24.4]	-91 [3.2]
LPBS	-123	-	-	-
HPBS	-125	-41 [28.6]	-90 [24.4]	-101 [1.0]
P3HT	-	235 [14.9]	199 [14.3]	-
LPBS:P3HT (99:1)	-123*	120 [10.2]**	98 [5.8]**	-
HPBS:P3HT (99:1)	-126	-41 [28.2]	-99 [14.3]	-95 [16.5]

Table S7. *Table showing the crystalline fractions (χ_c) estimations.*

Sample	χ_c of PDMS (weight)[a]		
	ΔH_c	*ΔH_{cc}*	*$\Delta H_{c\text{-}total}$*[b]
LPDMS-OH	0.33	0.26	0.59
HPDMS-OH	0.65	0.09	0.73
LPBS	-	-	-
HPBS	0.65	0.03	0.68
P3HT	-	-	-
LPBS:P3HT (99:1)	-	-	-
HPBS:P3HT (99:1)	0.38	0.44	0.82

[a]Calculated from $\chi_c = \Delta H / \Delta H_{100\%}$ [b]$\Delta H_{c\text{-total}} = \Delta H_c + \Delta H_{cc}$

11. Rheology

Rheology was carried out on a Bohlin Gemini Rheometer. Each sample was sandwiched between two parallel plates (20 mm) and kept at 25 °C using a Peltier heating system. The gap size for each sample was between 300-400 µm and each sample was left on the stage for 10 mins to allow for the sample to equilibrate. The amplitude strain of LPBS and HPBS was carried out at a frequency of 1 Hz, with increasing strain from 1 to 200%. The critical strain was then estimated to be the strain outside of the plateau of the elastic modulus (G') and the linear viscoelastic region (LVR) is the region where G' does not deviate by ±5% with increasing strain. The small angle oscillation strain (SAOS) experiments for all samples were carried out at a constant strain of 10% (below the critical strain for LPBS and HPBS), with increasing angular frequency from 0.1 to 50 Hz. The gradient of the viscous modulus (G'') and G' in the

terminal region was calculated by fitting a straight line and the error associated is due to the fit. The gradient was then compared to the power exponent in equation 3 and 4 in the main article.

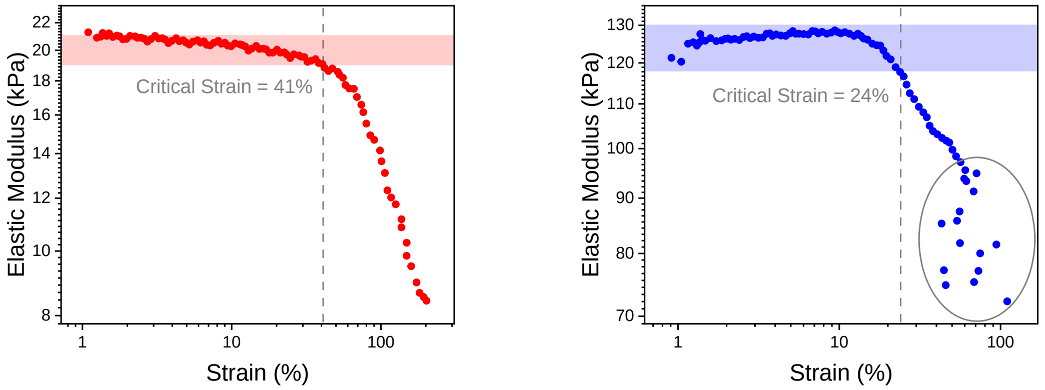

Figure S10. *Plot of strain amplitude of HPBS (right) and LPBS (left) at a constant frequency of 1 Hz. The grey dotted line indicates where the critical strain is, and the shaded area represents the ±5% deviation from the average of the plateau. The grey circles highlights that the large noise in LBPS at high strain is due to deformation of the sample.*

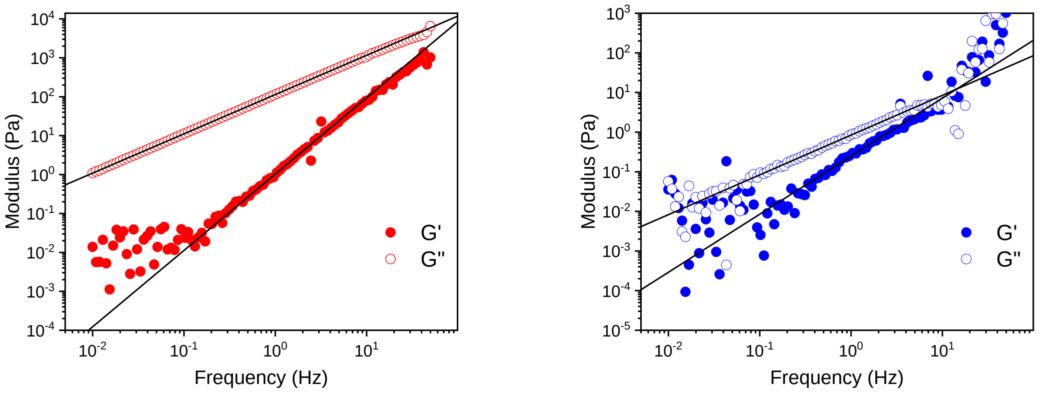

Figure S11. *Plot of modulus vs frequency for HPDMS-OH (left) and LPDMS-OH (right) obtained from the SOAS experiments at constant strain of 10 %, showing G' (closed circles) and G" (open circles). The black lines show the linear fit.*

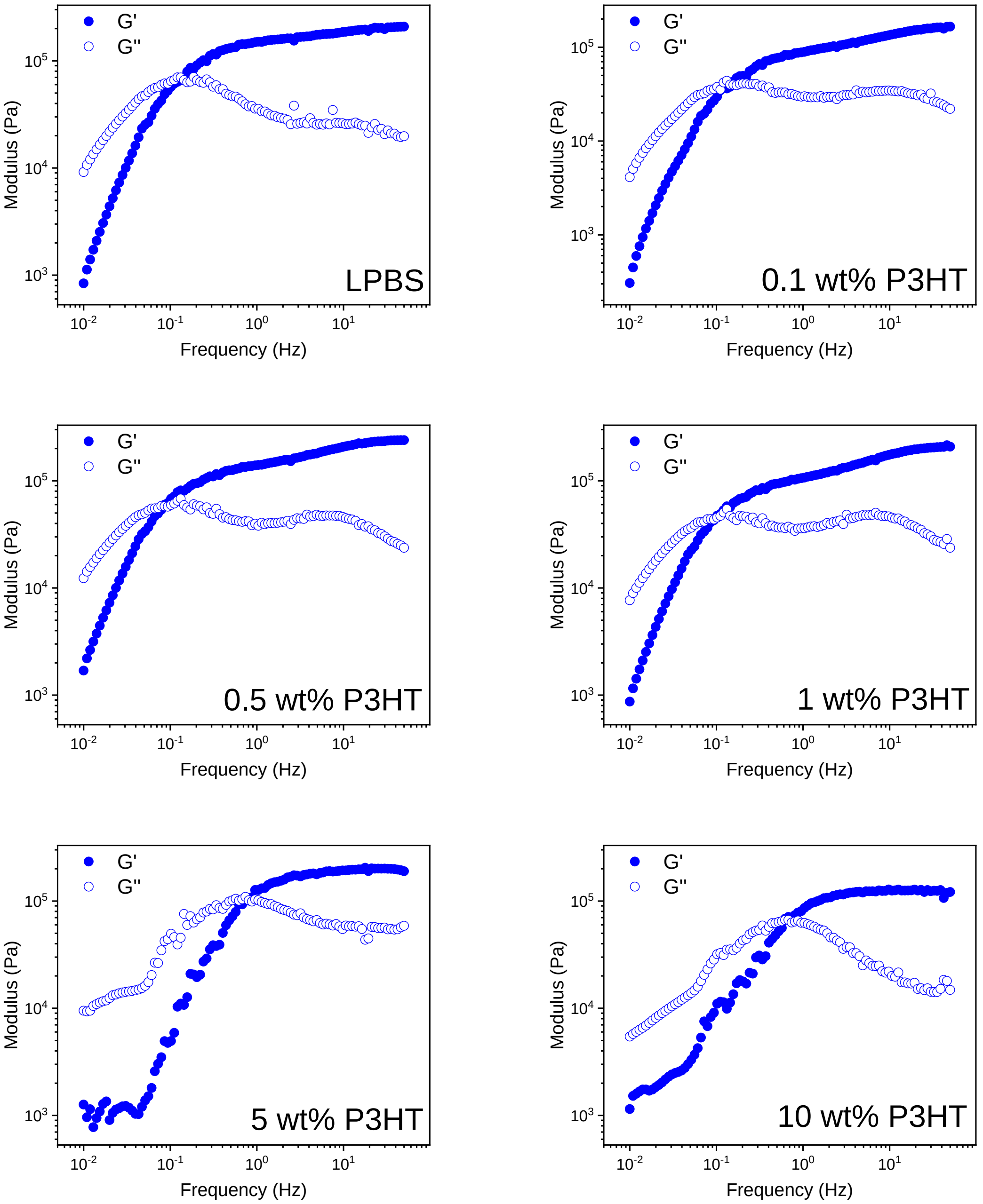

Figure S12. *Plot of modulus vs frequency for LPBS and each blend with P3HT obtained from the SOAS experiments at constant strain of 10 %, showing G' (closed circles) and G" (open circles).*

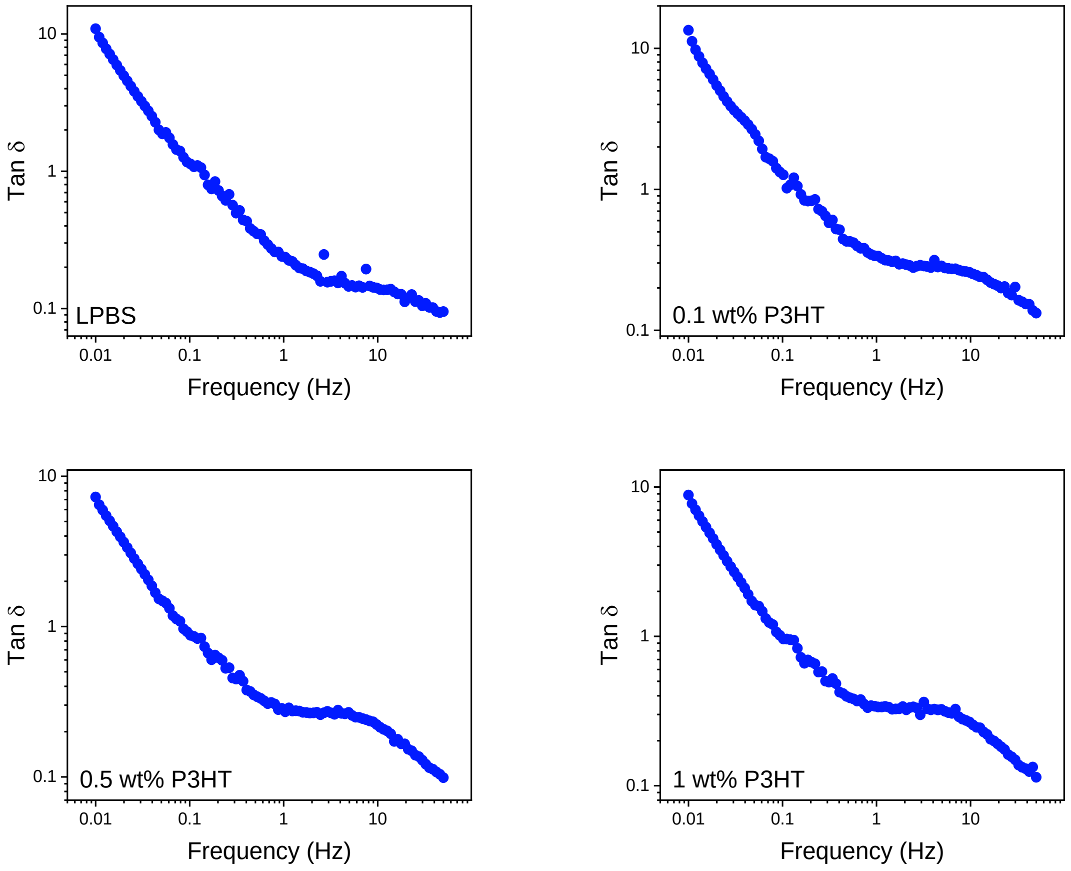

Figure S13. *Plot of Tan δ vs frequency for LPBS and 0.1-1 wt% P3HT.*

Table S8. *Table of the relaxation times and gradients in the terminal region extracting from the SOAS plots of for LPBS and its blends with P3HT.*

**P3HT Concentration in LPBS (wt%)**	**Relaxation Time (s)**		**Gradient in Terminal Region**[c]	
	Terminal[a], *τ*	*Transient*[b], τ_2	*G'*	*G"*
0.0	1.11	1.17×10^{-2}	2.03 ± 0.02	1.01 ± 0.02
0.1	1.20	1.64×10^{-2}	2.08 ± 0.06	1.09 ± 0.03
0.5	1.94	2.33×10^{-2}	1.83 ± 0.03	0.86 ± 0.03
1.0	1.64	2.44×10^{-2}	1.96 ± 0.03	0.94 ± 0.03
5.0	0.22	-	-	-
10.0	0.25	-	-	-

[a] Obtained from the frequency crossover point between G' and G". [b] Obtained from the maximum G" between 1 and 10 Hz. [c] Obtained from the gradient in the terminal region for G' and G". All fits have an R^2 = 0.99 and the error arises from the error of the line of best fit.

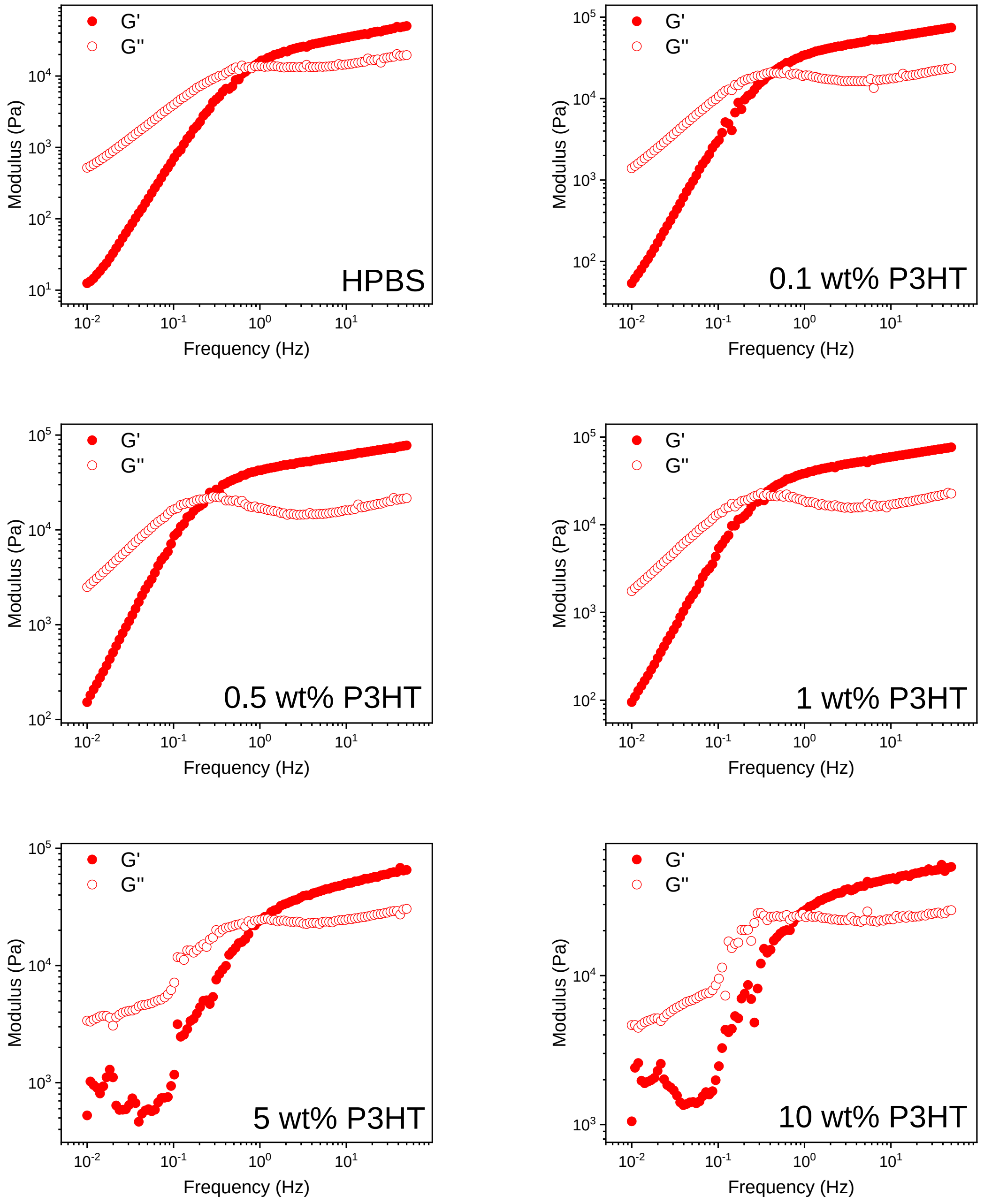

Figure S14. *Plot of modulus vs frequency for HPBS and each blend with P3HT obtained from the SOAS experiments at constant strain of 10 %, showing G’ (closed circles) and G” (open circles).*

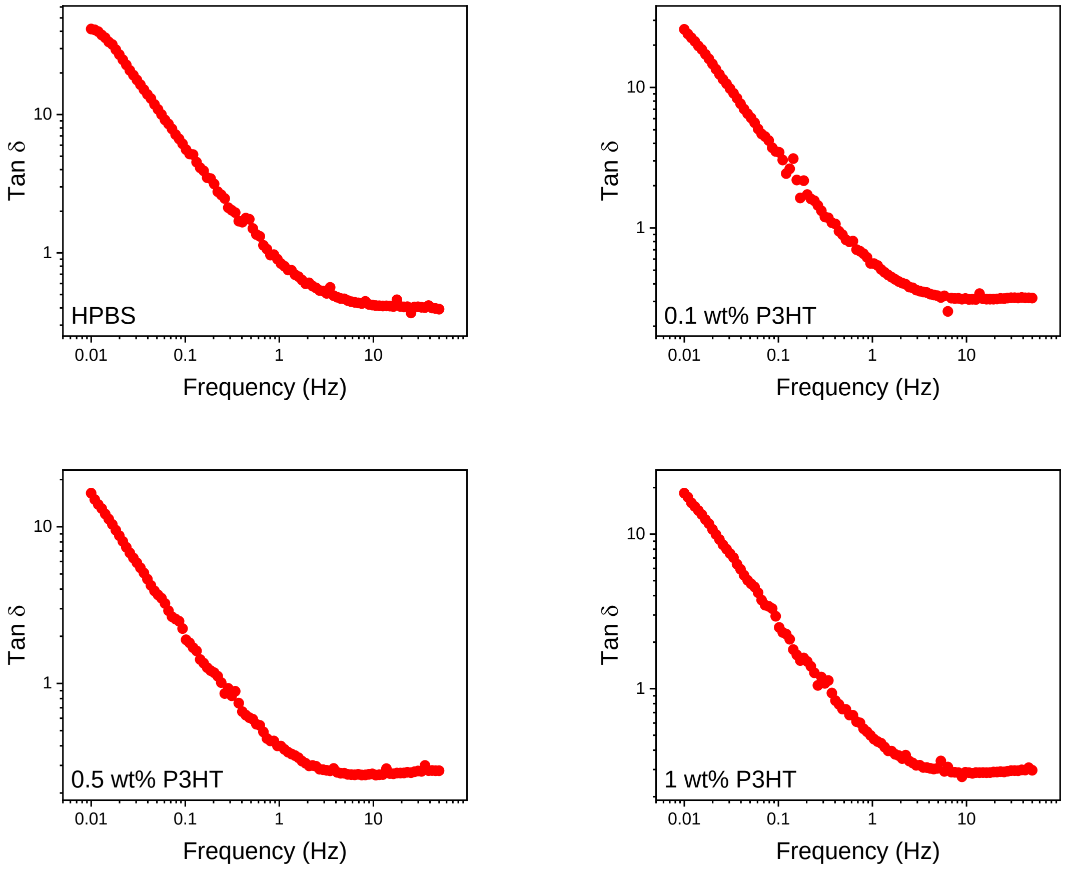

Figure S15. *Plot of Tan δ vs frequency for HPBS and 0.1-1 wt% P3HT.*

Table S9. *Table of the relaxation times and gradients in the terminal region extracting from the SOAS plots of for HPBS and its blends with P3HT.*

**P3HT Concentration in HPBS (wt%)**	**Relaxation Time (s)**		**Gradient in Terminal Region[c]**	
	Terminal[a], τ	*Transient[b]*, τ_2	*G'*	*G"*
0.0	0.19	-	1.83 ± 0.01	0.09 ± 0.01
0.1	0.37	-	1.81 ± 0.01	0.90 ± 0.01
0.5	0.64	-	1.75 ± 0.01	0.84 ± 0.01
1.0	0.51	-	1.73 ± 0.01	0.89 ± 0.01
5.0	0.16	-	-	-
10.0	0.19	-	-	-

[a] Obtained from the frequency crossover point between G' and G". [b] Obtained from the maximum G" between 1 and 10 Hz. [c] Obtained from the gradient in the terminal region for G' and G". All fits have an R^2 = 0.99 and the error arises from the error of the line of best fit.

12. References

[1] P. A. Finn, I. E. Jacobs, J. Armitage, R. H. Wu, B. D. Paulsen, M. Freeley, M. Palma, J. Rivnay, H. Sirringhaus, C. B. Nielsen, *Journal of Materials Chemistry C* **2020**, *8*, 16216-16223.

[2] M. Tang, W. T. Wang, D. H. Xu, Z. G. Wang, *Industrial & Engineering Chemistry Research* **2016**, *55*, 12582-12589.

[3] D. L. Liu, Z. C. Ding, Y. Wu, S. F. Liu, Y. C. Han, K. Zhao, *Macromolecules* **2022**, *55*, 297-308.

[4] M. Díaz de los Ríos, E. Hernández Ramos, *SN Applied Sciences* **2020**, *2*, 676.
